# The Potential Markers of Circulating microRNAs and long non-coding RNAs in Alzheimer's Disease

**DOI:** 10.14336/AD.2018.1105

**Published:** 2019-12-01

**Authors:** Yanfang Zhao, Yuan Zhang, Lei Zhang, Yanhan Dong, Hongfang Ji, Liang Shen

**Affiliations:** ^1^Institute of Biomedical Research, Shandong Provincial Research Center for Bioinformatic Engineering and Technique, Zibo Key Laboratory of New Drug Development of Neurodegenerative diseases, School for Life Science, Shandong University of Technology, Zibo, China.; ^2^Institute for Translational Medicine, Qingdao University, Qingdao, China.

**Keywords:** Alzheimer’s disease, circulating, miRNA, lncRNA

## Abstract

Alzheimer’s disease (AD) is a neurodegenerative disorder and one of the leading causes of disability and mortality in the late life with no curative treatment currently. Thus, it is urgently to establish sensitive and non-invasive biomarkers for AD diagnosis, particularly in the early stage. Recently, emerging number of microRNAs (miRNAs) and long-noncoding RNAs (lncRNAs) are considered as effective biomarkers in various diseases as they possess characteristics of stable, resistant to RNAase digestion and many extreme conditions in circulatory fluid. This review highlights recent advances in the identification of the aberrantly expressed miRNAs and lncRNAs in circulatory network for detection of AD. We summarized the abnormal expressed miRNAs in blood and cerebrospinal fluid (CSF), and detailed discussed the functions and molecular mechanism of serum or plasma miRNAs-miR-195, miR-155, miR-34a, miR-9, miR-206, miR-125b and miR-29 in the regulation of AD progression. In addition, we also elaborated the role of circulating lncRNA major including beta-site APP cleaving enzyme 1 (BACE1) and its antisense lncRNA BACE1-AS in AD pathological advancement. In brief, confirming the aberrantly expressed circulating miRNAs and lncRNAs will provide an effective testing tools for treatment of AD in the future.

Alzheimer’s disease (AD) is one of the most prevalent age-related neurodegenerative disorders and a leading cause of disability and mortality in the late life [1]. It is characterized by the pathological changes including the formation of intracellular neurofibrillary tangles, accumulation of amyloid-β (Aβ) peptides and Tau proteins, which lead to variable emotion alteration, personality changes, inappropriate social behaviors, progressive memory impairment and cognitive deficits, ultimately cause death [1-5]. With the accelerated process of aging society, aging related diseases especially AD bring a serious public health challenge. Six to eight in ten dementia patients have a diagnosis of AD [6]. An estimated 50 million people suffer from AD worldwide currently [6]. More seriously, the incidence of AD throughout the world is predicted to be 75.6 million by 2030 and 135.5 million by 2050, which is almost triple the existing population affected [7].

The cause and molecular mechanism of AD pathogenesis remain largely unclear due to AD is one of the most complicated and complex age-related disease. Recently, growing advancement has made in diagnosis and pharmacotherapy of AD, however, no effective cure and prevention measure is able to halt the disease development or reverse the brain alteration. The diagnosis of the disease is based on the history of symptoms, thus, reliable biomarker for early diagnosis is extremely pivotal for prevention the AD process. In this review, we summarized the correlation between the ectopic expressed microRNAs (miRNAs), long noncoding RNAs (lncRNAs) in circulatory fluid and AD patients (Fig. 1), which providing competent evidence for searching novel therapy targets and biomarkers for AD.

**Figure 1.**
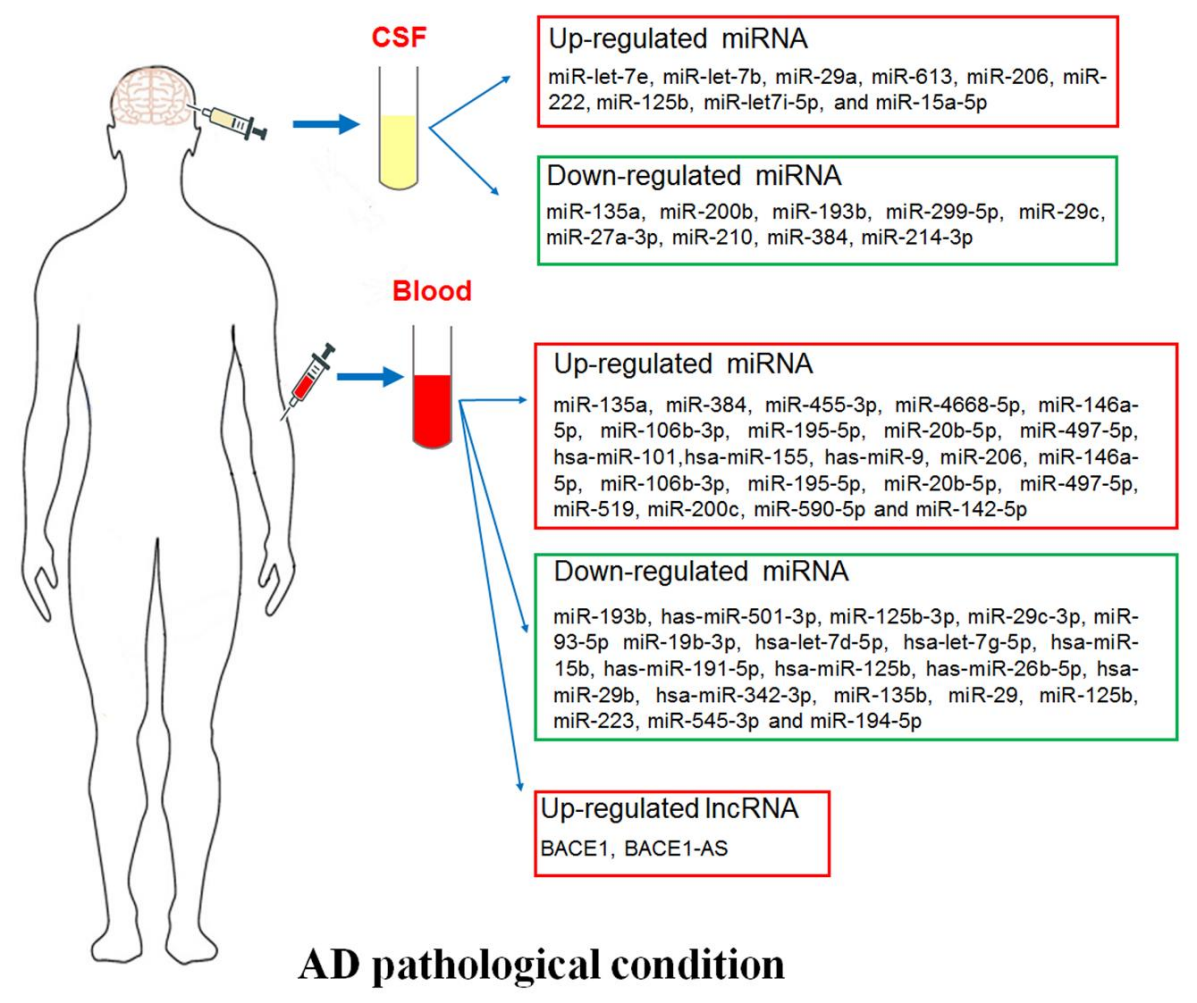
Circulating biomarkers in AD pathological condition. Plasma/serum biomarkers including miRNAs and lncRNAs, and cerebrospinal fluid (CSF) miRNAs.

## 1. Circulating miRNA and AD

### Circulating miRNA

miRNAs are a family of short non-coding RNAs with approximately 18~22 nucleotides, which suppress protein expression by binding the 3’untranslated region (3’UTR) of protein mRNA or promoting mRNA degradation [8]. Not surprisingly, miRNAs related pathways participate in various diseases including neurodegenerative diseases [9]. As one of major great challenges in biomarker analysis in central nervous system diseases, it is urgently to search a suitable, non-invasive and blood-based early biomarker for AD. Recently, circulating miRNAs have attracted more attention with several advantages to be good biomarkers. They are stable in the circulation, resistant to RNAase digestion and many extreme conditions including extreme pH, high temperature, extended storage, and multiple freeze-thaw cycles [10]. More importantly, miRNAs were able to be detected in all of the 12 body fluids [11], and the differential expression levels are tightly involved in various diseases or certain biological/pathological stages [12]. Here, blood and cerebrospinal fluid (CSF) miRNAs are introduced in this review as potential biomarkers in AD diagnosis.

### Blood miRNA and AD

It is a simple, affordable, less invasive or time saving detection method to explore the circulatory miRNAs in the blood as large-scale peripheral markers of patients with AD. A quantity of studies elaborated that the expression levels of miRNAs were checked in the blood of AD patients and normal controls at the same age (Fig. 1). The expression levels of miRNAs including miR-135a [13], miR-384 [13], miR-455-3p [14], miR-4668-5p [14], miR-146a-5p, miR-106b-3p, miR-195-5p, miR-20b-5p, and miR-497-5p [15], hsa-miR-101, hsa-miR-155, has-miR-9 [16], miR-206 [17], miR-146a-5p, miR-106b-3p, miR-195-5p, miR-20b-5p, miR-497-5p [15], miR-519 [18], miR-200c [19], miR-590-5p and miR-142-5p [20] were up-regulated, while miR-193b [13], has-miR-501-3p [21], miR-125b-3p, miR-29c-3p, miR-93-5p, miR-19b-3p [15], hsa-let-7d-5p, hsa-let-7g-5p, hsa-miR-15b, has-miR-191-5p, has-miR-26b-5p, hsa-miR-29b, hsa-miR-342-3p [16], miR-135b [22], miR-29, miR-125b [16, 20], miR-223 [18], miR-545-3p [23] and miR-194-5p [20] were down-regulated in serum of AD patients compared with that of normal subjects. In addition, serum miR-206 was enhanced in the individuals with amnestic mild cognitive impairment tend to progress to AD [17]. Furthermore, circulating miR-28-3p level was elevated but expressions of miR-125b, miR-9 and miR-191-5p were reduced in the APP/PS1 transgenic mouse model of AD [24]. Next, we detailed introduced several serum miRNAs which were studied more in AD progression.

### miR-195 and AD

The serum level of miR-195 is elevated in AD patients [15]. Zhang *et al* reported that decreased expression of mitofusin-2 (mfn2) was linked with mitochondrial dysfunction during the progression of AD, which was considered as mfn2 level being partly modulated by miR-195 [25]. Discordantly, other studies identified miR-195 as a negative modulator in AD advancement. The formation of Aβ plaques is one of the crucial pathological hallmarks of AD [26-28]. Aβ peptide is produced from amyloid precursor protein (APP) which is cleavage by beta-site APP cleaving enzyme 1 (BACE1) [26]. The level of BACE1 was reduced by miR-195 via its binding site targeting BACE1 3’UTR, and down-regulation of miR-195 resulted amyloidogenesis in senescence-accelerated mice (SAMP8) or in chronic brain hypoperfusion rats with bilateral common carotid artery occlusion [27, 28]. On the basis, abnormal expression level of miR-195 promotes the pathological process of AD.

### miR-155 and AD

Neuroinflammation and immune-defense are considered as key factors in AD progression and pathogenesis [29]. MiR-155 is one of the most well studied immune-related miRNAs in AD-related neuroinflammatory events. Persistent microglial activation is able to initiate neuronal damage and eventually causes AD [30]. MiR-155 level was remarkably increased in SH-5Y5Y cells transfected with Swedish mutant of APP_695_ accompanying with higher APP and Aβ_1-40_ production and enhanced inflammatory marker expressions [31]. A highly expression level of miR-155 was also confirmed in 3xTg AD animal model [32]. This early up-regulated miR-155 and c-Jun simultaneously accompanied with an enhanced activation of microglia and astrocyte, thus triggered the production of inflammatory mediators [32]. Moreover, dipeptidyl vinyl sulfone (VS) reduced Aβ-induced microglia activation via suppression the expression of inflammatory mediators, as well as prevention the elevated expression levels of miR-155 and miR-146a upon Aβ treatment [30]. miR-155 was also observed differentially expressed in blood-derived monocytes and monocyte-derived macrophages which were isolated from blood of AD, mild cognitive impairment (MCI) patients and healthy controls [33]. Lipopolysaccharide (LPS) treatment can lead to peripheral- and neuro- inflammation [34]. The recipient mice that received serum-derived exosomes from LPS-challenged mice showed characteristics of enhanced microglial activation, elevated pro-inflammation cytokine and its mRNA production, especially increased the miR-155 expression level [35]. In addition, miR-155 contributed to regulation of AD disease via activation of diverse of T cells functions during inflammation which may alleviated AD related severe pathologies [36]. On the basis, as a key inflammation and immune related miRNA, miR-155 exerts a positive function in promoting screening effective treatments for AD.

### miR-34a and AD

The expression of peripheral miR-34a was dramatically up-regulated in AD subjects compared to normal elderly controls [16, 37]. MiR-34a was also highly expressed in specific brain regions of AD patients, 3xTg-AD mouse model as well as cerebral cortex of APPswe/PS mice [38-40]. Moreover, the enhanced miR-34a expression in brain compared to age matched healthy control was closely associated with severity of AD pathology [38]. P53 is a crucial response element of miR-34a, p53/miR-34a axis promotes cell apoptosis via activating caspase-3 and suppressing Sirt1 and Bcl2 expressions in AD transgenic mice brain [39, 41]. Another p53-family member Tap73 (p73) drives miR-34a expression through binding specific sites of miR-34a promoter. A remarkable raised miR-34a/p73 expression was found in AD hippocampus, which participated in modulating synaptic activity by lessening synaptotagmin-1 expression in brain from AD patients [42]. In addition, knockout of miR-34a in APP/PS1 mice decreased Aβ plague production and improved cognitive function by depression of γ-secretase activity [40]. Thus, miR-34a is considered as a key modulator in process of AD pathology.

### miR-9 and AD

The declined level of whole-blood has-miR-9-5p was tightly linked with a raised risk of AD [24, 43]. In Aβ treated hippocampal cells, as well as in APP23 transgenic mice or human AD cortex, miR-9 and miR-181c were down-regulated and exerted their roles in brain homeostasis via targeting TGFBI, TRIM2, SIRT1 and BTBD3 [44]. Aβ_42_ treatment initiated CAMK2-AMPK signaling activation and synaptotoxic impairment as the result of the reduced expression of miR-9, while up-regulation of miR-9 was sufficient to restore Aβ_42_-induced dendritic spine loss [45]. Thus, as a drug treatment target, osthole exerts its powerful neuroprotective effect against AD via promoting miR-9 level, reducing CAMKK2 and p-AMPKα expressions and subsequently suppressing the Notch signaling pathway [46, 47]. Hence miR-9 was a neuroprotective regulator in AD development.

### miR-206 and AD

The level of serum miR-206 and miR-132 were elevated in MCI patients compared with age-matched normal controls. Combining detection of miR-206 and miR-132 achieved a highest areas under curves (AUC), which is an index of miRNA’s diagnostic performance [48, 49]. Yet, the serum miR-206 level was increased in amnestic MCI (aMCI)-AD patients than aMCI-aMCI group whereas no notable differences in serum levels of miR-132 [17]. The miR-206 levels were also up-regulated in the Tg2576 mice brain and the temporal cortex of human AD brains [50], the hippocampal tissue and plasma of embryonic APP/PS1 transgenic mice [51]. The enhanced expression of miR-206 major promoted the detrimental effect of Aβ_42_ on brain-derived neurotrophic factor (BDNF) via inhibiting the level of BDNF [50, 51]. Further, miR-206 inhibitor is able to relive the detrimental effects of Aβ_42_, and it is a target of donepezil, a drug approved for treating AD in clinic [52]. Therefore, miR-206 was a modulator to exacerbate the AD advancement.

### miR-125b and AD

The serum miR-125b was down-regulated in AD patients compared with that of control subjects [15, 53-55]. In addition, decreased circulating miR-125b was also found in the APP/PS1 transgenic mouse models of AD [24]. In the Aβ pathological condition, the reduced expression of miR-125b is a critical event for the neurotoxic effect in cortical neurons. 17β-estradiol can protect neurons from the Aβ-peptide caused neurotoxicity via up-regulation miR-125b expression [56]. However, it is inconsistently with the protection role of miR-125b in AD. MiR-125b was found highly expression in AD patients [57]. Up-regulation of miR-125b caused tau hyperphosphorylation, inhibited cell proliferation, promoted apoptosis, induced inflammation and oxidative stress by activation of CDK5 and p35/25, p44/42-MAPK signaling pathway, and suppressed expressions of forkhead box Q1 (FOXQ1), anti-apoptotic factor Bcl-W, and sphingosine kinase 1 (SphK1) [57-59]. It is probably that the aberrant expression of miR-125b contributes to the neural dysfunction in AD brain.

### miR-29 and AD

The serum of miR-29 expression was remarkably depressed in AD patients verse control subjects [15, 60-62]. The major events associated with the aberrant expression of miR-29 up-regulated the Aβ precursor protein BACE1 expression and subsequent caused Aβ accumulation [62, 63]. MiR-29c directly targets the 3’UTR of BACE1 mRNA [63]. Overexpression of miR-29 was able to reduce the level of BACE1 and Aβ accumulation in vitro, and ameliorate learning and memory in SAMP9 mice partially through enhancing the activity of protein kinase A/cAMP response element-binding protein [62, 63]. Thus, its highly pure and biologically active pre-miR-29b deliver using polyplexes to N2a695 cells can reduce BACE1 expression and Aβ_42_ level, which was considered as a potentially therapy way for AD [61]. In AD brains, neuron navigator 3 (NAV3) was highly expressed as the result of the attenuated miR-29a and miR-29c expressions, which also referred to dysregulation of axon guidance [64, 65]. The up-regulated expression of miR-29c-3p and miR-29b also exhibited neuroprotective functions in AD via targeting signal activators of transcription 3 (STAT3) and specificity protein 1 (Sp1), respectively [15, 60]. Thus, miR-29 family are potentially biomarkers for AD treatment.

## 2. CSF miRNAs and AD

Cerebrospinal fluid is a continuum of the brain, which is an attractive source of biomarkers reflecting central neuropathological features of the brain diseases including AD [66]. Recently, emerging studies have suggested that CSF contains circulating miRNAs, which were critical biomarkers with a high predictive accuracy in the pathogenesis process of AD (Fig. 1). The expression levels of miR-let-7e [67], miR-let-7b [67, 68], miR-29a [69], miR-613 [70], miR-206 [51], miR-222 [71], miR-125b [71], miR-let7i-5p [20] and miR-15a-5p [20] were increased, while miR-135a [72], miR-200b [72], miR-193b [73], miR-299-5p [74], miR-29c [20, 75], miR-27a-3p [76], miR-210 [77], miR-384 [78] and miR-214-3p [79] were decreased in the CSF from AD patients compared to healthy controls. The elevated miR-let-7b level in CSF from AD patients mainly originated from CD4+ T lymphocyte and was associated with neurotoxicity and t-tau/p-tau expression [67, 68]. miR-613 or miR-206 was responsible for the AD pathology via suppression the neuroprotector-BDNF [51, 70], which also accompanied with a decreased expression of miR-29c [75]. Under AD pathological conditions, the expressions of miR-384, miR-135a and miR-200b in CSF were declined, which attenuated their repression roles on the APP and BACE1 levels. Meanwhile, Aβ_42_ could also restrain miR-384 and miR-200 expression. The above may generated a vicious cycle resulted in accumulation of Aβ_42_ [72, 78]. Moreover, miR-193b was negatively correlated with Aβ_42_ in the CSF of dementia of Alzheimer-type (DAT) patients [73]. The low level of miR-27a-3p was linked with the enhanced tau but decreased Aβ levels [76] in the CSF from AD patients. The expressions of miR-299-5p and miR-214-3p were reduced upon AD conditions and resulted in autophagy by disinhibition of Atg5, LC3βII and Beclin1 levels, respectively [73, 79]. MiR-210 expression was abated in the CSF and serum accompanied with the decreased level of VEGF, which were associated with the severity of the AD [77]. With the thorough studies on CSF miRNAs in AD, they will be used as biomarkers to assess disease progression and therapeutic efficacy.

## 3. Circulating lncRNAs and Alzheimer’s disease

Recently, lncRNAs are also found in circulating fluid and play pivotal roles in various diseases [80]. LncRNAs are classically defined as > 200 nucleotides transcripts lacking protein-coding ability with biological regulatory and modificatory functions [81, 82]. As advanced transcriptome-wide profiling approach, emerging number of lncRNAs were comprehensively identified dysregulated in AD pathological brains [83, 84]. Circulating lncRNA was also described to participate in the occurrence and development of AD (Fig. 1) [85]. The lncRNA BACE1 level was dramatically up-regulated in AD patient’s plasma compared with normal control subjects, while no significantly alteration of plasma lncRNA 17A, 51A and BC200. Accordantly, Manzine *et al* also confirmed that plasma BACE1 level was elevated in AD patients [86]. However, Marison *et al* proved that there was no dramatically difference of BACE1 expression in blood between control, AD and non-AD neuropathology’s individuals. Interestingly, it was still considered as a potential marker used in diagnosis as that have somehow been involved with AD or AD-related elements [87]. In the context, we have introduced that BACE1 is a key modulator and its mediated the production of Aβ from APP is the rate limiting step in AD progression. BACE1 is also positively regulated by its antisense transcript (lncRNA BACE1-AS) [88], which partly binds with BACE1 mRNA and promotes BACE1 expression [89]. LncRNA BACE1 and BACE1-AS were checked highly expressed in the blood in brain related disease [90]. They promoted AD pathogenesis via enhancing Aβ and APP level and they were able to participate in learning and memory impairment via being stabilized by the primarily neuronal RNA-binding protein HuD in AD advancement [89]. In addition, the abnormal expressed BACE1 can be not only a biomarker for AD diagnosis, but also a therapeutic target by systemic delivery of BACE1 siRNA and its inhibitors [91, 92]. In brief, the lncRNAs in circulatory fluid from AD patients need to be intensively studied for supplying novel biomarkers or providing new drug treatment targets in AD development in the future.

## 4. Conclusions

The dementia related diseases are difficult to be definitive diagnosed. They are traditionally based on the history of the disease, the pattern of cognitive impairment, and on additional parameters evaluated via clinical examination, including blood tests and brain structural imaging, to exclude nondegenerative causes of the symptoms [93]. It is necessary and urgent to seek novel simple and effective biomarkers for diagnosis specific forms of dementia earlier, also in the pre-dementia stages of the disease, and with more specificity. As the increasing number of circulating miRNAs and lncRNAs are being to be reported abnormally expressed in AD patients. Characterizations of these AD-associated circulating miRNAs and lncRNAs offer the possibility of providing new insights into disease pathogenesis. In consideration of that non-coding RNAs in circulatory fluids are easily accessible and relatively stable, it is more reality to make a correct clinical prognosis for AD. Thus, confirming the aberrant expressed circulating miRNAs and lncRNAs will provide an effective testing tools for treatment of AD in the future.
